# Molecular Characterization of Putative Virulence Determinants in *Burkholderia pseudomallei*

**DOI:** 10.1155/2014/590803

**Published:** 2014-08-11

**Authors:** Suat Moi Puah, S. D. Puthucheary, Jin Town Wang, Yi Jiun Pan, Kek Heng Chua

**Affiliations:** ^1^Department of Biomedical Science, Faculty of Medicine, University of Malaya, 50603 Kuala Lumpur, Malaysia; ^2^Department of Medical Education, Research and Evaluation, Duke-NUS Graduate Medical School Singapore, 8 College Road, Singapore 169857; ^3^Department of Microbiology, National Taiwan University College of Medicine, Section 1, Jen-Ai Road, Taipei 10051, Taiwan; ^4^Department of Internal Medicine, National Taiwan University Hospital, Taipei 10051, Taiwan

## Abstract

The Gram-negative saprophyte *Burkholderia pseudomallei* is the causative agent of melioidosis, an infectious disease which is endemic in Southeast Asia and northern Australia. This bacterium possesses many virulence factors which are thought to contribute to its survival and pathogenicity. Using a virulent clinical isolate of* B. pseudomallei* and an attenuated strain of the same* B. pseudomallei* isolate, 6 genes* BPSL2033, BP1026B_I2784, BP1026B_I2780, BURPS1106A_A0094, BURPS1106A_1131*, and *BURPS1710A_1419* were identified earlier by PCR-based subtractive hybridization. These genes were extensively characterized at the molecular level, together with an additional gene* BPSL3147* that had been identified by other investigators. Through a reverse genetic approach, single-gene knockout mutants were successfully constructed by using site-specific insertion mutagenesis and were confirmed by PCR.* BPSL2033::Km* and* BURPS1710A_1419::Km* mutants showed reduced rates of survival inside macrophage RAW 264.7 cells and also low levels of virulence in the nematode infection model.* BPSL2033::Km* demonstrated weak statistical significance (*P* = 0.049) at 8 hours after infection in macrophage infection study but this was not seen in* BURPS1710A_1419::Km*. Nevertheless, complemented strains of both genes were able to partially restore the gene defects in both *in vitro* and *in vivo* studies, thus suggesting that they individually play a minor role in the virulence of* B. pseudomallei*.

## 1. Introduction

*Burkholderia pseudomallei* is a Gram-negative motile bacillus, that is, a facultative anaerobe and an environmental saprophyte. It is readily recovered from the soil and surface waters in endemic areas, that is, Southeast Asia and Northern Australia [1]. This bacterium possesses a remarkable capacity to infect humans and animals, causing melioidosis which is an important cause of sepsis in the tropics [2]. It has been considered a potential bioweapon and so virulence factors that correspond to its pathogenesis are being intensively studied at an increasing rate.

The availability of complete genome sequence database of organisms allows researchers to discover many molecules and mechanisms that may be involved in the virulence of* B. pseudomallei* [3, 4]. Several approaches, including subtractive hybridization [5], comparative genomics [6], signature-tagged mutagenesis [7], transposon mutagenesis [8],* in vivo* expression technology [9], microarray [10], and computational methods [11, 12], have accelerated the discovery of virulence factors over the past decades. The availability of the genomic sequences of several* B. pseudomallei* strains has rapidly added candidate virulence genes to databases.

In general, past studies have focused on genomic differences between species, that is, the virulent* B. pseudomallei* and a closely related but avirulent family member* B. thailandensis* [5, 6]. PCR-based subtractive hybridization was recently undertaken in our laboratory using a virulent clinical isolate* B. pseudomallei (v)* and an attenuated strain of the same* B. pseudomallei* isolate (*av*) [13]. PCR-based subtractive hybridization successfully demonstrated 6 subtracted DNA fragments that were unique to the virulent strain of* B. pseudomallei*, whereas these DNA fragments were not seen in its “attenuated” strain [13]. Sequencing of the subtracted DNA fragments revealed 6 unique genes with unknown functions as follows:* BPSL2033, BP1026B_I2784, BP1026B_I2780, BURPS1106A_A0094, BURPS1106A_1131, and BURPS1710A_1419.*

Besides the 6 putative “virulence” determinants,* BPSL3147* is another potential virulence determinant identified by Cuccui et al. [7]. This gene encodes a putative lipoprotein and is reported to be a putative lipoprotein containing 39.16% amino acid that is identical to a* VacJ* lipoprotein in* Shigella flexneri*. The Tn10 mutant of* S. flexneri* YSH6000T* VacJ* lipoprotein was unable to spread from cell to cell, suggesting* VacJ* is important for intercellular spread of the organism [14].

The aim of this study was to extensively characterize the 6 putative virulence determinants which were absent in the “attenuated” strain (*av*) that is believed to have reduced virulence that occurred after several subcultures and long-term storage in the laboratory, together with an additional candidate,* BPSL3147,* using the same methodology, that is, gene knockout approach, using* in vitro* and* in vivo* assays.

## 2. Material and Methods

### 2.1. Bacterial Strains, Media, and Culture Conditions

Bacterial strains and plasmids used are listed in Table 1. The clinical* B. pseudomallei *strain was isolated from the blood of a patient CMS, at the University Hospital, University of Malaya, Kuala Lumpur, who died from melioidosis as described in previous report [11] and this strain was used throughout the study (henceforth referred to as Bp-CMS). The* Escherichia coli* strains DH10B, CC118*λ*pir, S17-1*λ*pir, vector pUT-Km, and chloramphenicol acetyltransferase (CAT) cassette were obtained from Prof. Dr. Wang Jin-Town (National Taiwan University, Taiwan). All strains were grown in Luria-Bertani (LB) medium at 37°C and, when appropriate, antibiotics were used at the following final concentrations: ampicillin 100 *μ*g/mL, kanamycin 50 *μ*g/mL, chloramphenicol 100 *μ*g/mL, and streptomycin 100 *μ*g/mL. The mouse leukaemic monocyte macrophage cell line RAW264.7 was obtained from American Type Culture Collection (ATCC, USA). It was cultured and maintained in flasks (Corning, USA) with DMEM (Gifco, USA) supplemented with 10% (v/v) fetal bovine serum (Gifco, USA), 4 mM L-glutamine, and an antibiotic mixture containing 100 U/mL penicillin and 0.1 mg/mL streptomycin at 37°C in 5% CO_2_.

### 2.2. Construction of Mutants

Genes* BPSL2033*,* BP1026B_I2784*,* BP1026B_I2780*,* BURPS1106A_A0094*,* BURPS1106A_1131*,* BURPS1710A_1419,* and* BPSL3147* were disrupted by homologous recombination using a suicide vector pUT-Km [15, 16]. Briefly, a partial region of the gene to be inactivated (serving as a significant site of gene exchange through conjugation and recombinant events) was amplified by polymerase chain reaction (PCR) using primer pairs as listed in Table 2. The amplicon was subcloned into pGEM-T easy vector, digested with EcoRI, and subcloned into the suicide vector pUT-Km [15]. Selection of transformants by plating onto LB agar containing kanamycin and rapid screening for desired inserts by colony PCR was performed using primers KmF and KmR. The construct was then transformed into* E. coli* S17-1*λ*pir and introduced into the wild-type* B. pseudomallei* (Bp-CMS) by conjugation. An insertion mutant was selected using LB agar supplemented with kanamycin and site-specific chromosomal integration was verified by PCR using vector- and corresponding gene-specific primer pairs.

### 2.3. Construction of Complemented Plasmids

Intact genes* BURPS1710A_1419, BPSL2033, BP1026B_I2784, BP1026B_I2780, BURPS1106A_A0094, BURPS1106A_1131*, and* BPSL3147* and their promoters were amplified by PCR and cloned into a pGEM-T-CAT plasmid. These complemented plasmids were then reintroduced into the corresponding insertion mutants by transformation. The resulting complemented strains were selected using LB agar containing chloramphenicol.

### 2.4. Bacterial Growth Curve

The growth of* B. pseudomallei* strains in LB broth was monitored over 8 h by taking 1 mL of culture broth every hour to perform OD600 readings. The wild-type Bp-CMS was used as a positive control for growth in LB.

### 2.5. Bacterial Replication Assays

Intracellular bacterial survival and replication was assayed in the mouse macrophage-like cell line RAW 264.7. Cells were seeded at a density of 1 × 10^5^ cells/well into 24-well culture plates (Corning, USA) and incubated overnight at 37°C in 5% CO_2_. An overnight culture of* B. pseudomallei* strain was diluted 1 : 100 and grown at 37°C for 3 h with shaking to reach mid-log phase. Cell monolayers were then washed twice with PBS and incubated in fresh DMEM without antibiotic for 1 h prior to infection with bacteria. The bacterial suspension was added at a MOI of 100 : 1 and the coculture was immediately centrifuged at room temperature at 170 ×g for 5 min to bring the bacteria in direct contact with the host cells. After 1 h, the cells were washed twice with PBS and incubated in fresh DMEM containing 300 *μ*g/mL of tetracycline to suppress the growth of residual extracellular bacteria. Tetracycline was used instead of gentamicin, as Bp-CMS is resistant to gentamicin. At 2, 4, and 8 h after infection, infected monolayers were washed twice with PBS and lysed with 0.1% (v/v) Triton X-100 for 15 min. Serial dilutions of the released bacteria (expressed as colony forming units CFU) were plated on tryptic soy agar (TSA) plates to enumerate bacterial loads by direct colony counts. The number of internalized bacteria obtained at 2 h after infection represented the initial entry of bacteria, whereas at 4 and 8 h after infection represented intracellular bacterial replication. Bacterial survival was normalized to counts obtained at 2 h after infection and the relative survival rate presented as a percentage.

### 2.6. Virulence Testing with* C. elegans*

The wild-type* C. elegans* N2 was obtained from Carolina Biological Supply Company (USA). The nematode was propagated on nematode growth medium (NGM) plate and fed on the normal food source* E. coli* OP50-1, which was a kind gift from Prof. Dr. Sheila Nathan (National University of Malaysia, Malaysia). Killing assays were performed as previously described by Tan et al. [17] with minor modifications. All nematodes were age-synchronized by a bleaching procedure prior to the killing assay [18]. All* B. pseudomallei* derived strains (wild-type Bp-CMS, 7 insertion mutants, and 7 complemented strains) and* E. coli* OP50-1 were grown overnight at 37°C and 40 *μ*L of each culture was spread on NGM plates containing 50 *μ*g/mL 5-fluorodeoxyuridine (Merck, USA) to inhibit the eggs of* C. elegans* from hatching. Plates were incubated at 37°C for 24 h and then allowed to equilibrate for 24 h at room temperature before coculturing with the host worms. Thirty age-matched hermaphrodites were individually transferred to freshly lawned plates by using the flattened tip of a worm pick (platinum wire). The plates were incubated at room temperature and virulence was tracked by counting the number of live and dead worms every 24 h for 3 days. Three independent experiments were carried out for each strain and each test was performed in triplicate (with a total of 270 worms). A worm was considered dead on failure to respond to gentle touch by the worm pick.* E. coli* OP50-1 was used as a negative control. The resulting data was analyzed with GraphPad Prism 5 software and plotted using the Kaplan-Meier survival plot.

## 3. Results

### 3.1. Insertion Mutant Construction and Growth

**Figure 1:**
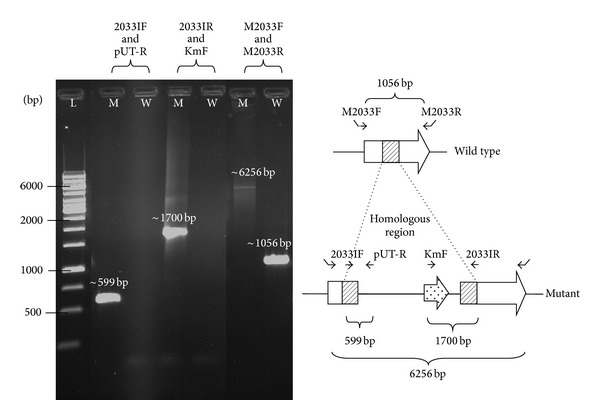
Representative of mutant construct. Verification of the construction of* BPSL2033::Km* mutant by PCR using specific alignment primers. M and W represent mutant and wild-type strains, respectively, while L indicates 1** **kb DNA ladder from Fermentas (USA).

Positive chromosomal integration mutants were successfully constructed for the 7 candidate genes, on the first or second attempt, and a representative of construct is shown in Figure 1. All 7 mutant strains demonstrated similar growth rates to the parental strain in liquid media over 8 h.

### 3.2. Bacterial Replication and Survival Assay

Overall, wild-type bacteria were able to survive and replicate in macrophage cells over the course of the experiments. In contrast, the 5 mutant strains* BPSL2033::Km, BP1026B_I2780::Km, BURPS1106A_A0094::Km, BURPS1710A_1419::Km*, and* BPSL3147::Km* showed reduced intracellular survival inside RAW264.7 cells at 4 and 8 h post infection, but only* BPSL2033::Km* reached statistical significance (*P* = 0.049) at 8 h post infection. Another 2 mutant strains* BP1026B_I2784::Km* and* BURPS1106A_1131::Km* demonstrated no difference in survival in RAW264.7 cells at 4 and 8 h post infection.

**Figure 2:**
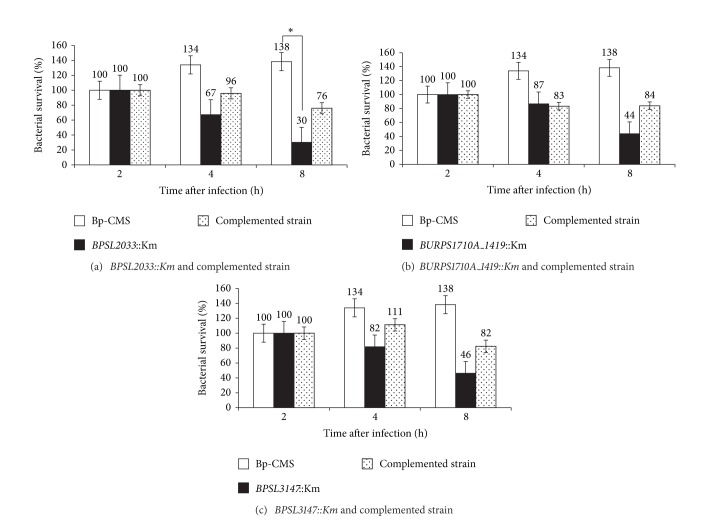
Bacterial survival and replication within RAW264.7 macrophage-like cells infected at MOI of 100 using wild-type strain and the insertion mutants as well as their complemented strains. Data represents means and standard errors of 3 separate experiments; each experiment was carried out in 3 technical replicates for each time point. Asterisk indicates significant differences (*P* < 0.05) relative to the wild-type strain Bp-CMS at each time point.

Most of these plasmid-complemented strains partially restored intracellular survival and replication except for* BP1026B_I2780::Km, BURPS1106A_A0094::Km*, and* BURPS1106A_1131::Km*. The results suggest that 3 genes,* BPSL2033::Km* (*P* = 0.049),* BURPS1710A_1419::Km* (*P* = 0.165), and* BPSL3147::Km* (*P* = 0.076), individually had little effect on the intracellular survival of* B. pseudomallei* in phagocytic cells (Figures 2(a) to 2(c)).

### 3.3. *Caenorhabditis elegans* Killing Assay

**Figure 3:**
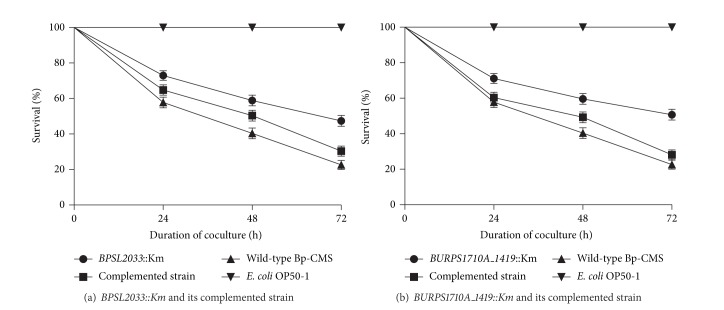
Kaplan-Meier survival curves for* C. elegans* infected with different strains of* B. pseudomallei*. Values are the pooled data from triplicate of 3 separate experiments (*N* = 270) (*E. coli* OP50-1 was the negative control).

The 6 mutants (*BPSL2033::Km, BP1026B_I2784::Km, BP1026B_I2780::Km, BURPS1106A_1131::Km, BURPS1710A_1419::Km*, and* BPSL3147::Km*) exhibited low levels of attenuated virulence in* C. elegans*, where survival rates of worms were only 2-fold higher than that of the wild type (data not shown). Among them, the most attenuation of virulence was observed in* BURPS1710A_1419::Km* as 51% worms were able to survive, followed by* BPSL2033::Km* (47%) compared to the wild-type parental strain (23%) after 3 days (Figures 3(a) and 3(b)). The complemented strains of both mutants,* BURPS1710A_1419::Km* and* BPSL2033::Km*, showed at least partial restoration of virulence, thus suggesting a minor role in the nematode infection model.

## 4. Discussion

There was no difference in the growth rates of all 7 mutants compared to that of the wild type when assayed in rich media (data not shown), ruling out the possibility that these genes are not affecting the growth but involved in other aspects such as pathogenicity. The infection assay on phagocytic cells showed that, without the presence of gene* BPSL2033* (putative transport-related membrane protein), the mutants demonstrated reduced ability to replicate and survive over 8 h. This result was supported by a plasmid-encoded complemented strain, which demonstrated partial restoration of intracellular survival when compared to wild-type Bp-CMS. Similar outcomes were seen with* BURPS1710A_1419* (putative lipoprotein) and* BPSL3147* (lipoprotein).

At 8 h post infection, loss of gene* BPSL2033* showed a 5-fold reduction in survival time in RAW264.7 cells while both* BURPS1710A_1419* and* BPSL3147* resulted in a 3-fold reduction in survival, indicating that these genes may be involved in intracellular survival. There was a weak but still significant difference (*P* = 0.049) exhibited by* BPSL2033::Km* compared to Bp-CMS. Thus,* BPSL2033* may act in concert with other genes and play an essential role in virulence. Subtractive hybridization, as reported in our earlier study, demonstrated that Bp-CMS contained 6 DNA sequences that were not found in the attenuated strain, indicating that maximal virulence probably requires multiple genes acting together in concert [13]. This hypothesis is further supported by the present study in which* B. pseudomallei* virulence in the phagocytic cell line model was not critically dependent on any single putative gene tested.

Several studies have suggested that a double mutant and not a single mutant of* B. pseudomallei* contributed significantly to the growth inside murine macrophage [19, 20]. Future experiments involving the use of double mutants (i.e.,* BPSL2033* and* BURPS1710A_1419*) may prove this possibility. In the context of* BPSL3147*, there may have been other unidentified gene(s) acting together for full virulence in* B. pseudomallei* infections. It is unclear why these genes* BPSL2033*,* BURPS1710A_1419,* and* BPSL3147* with their corresponding complemented plasmids only restored intracellular survival and replication to approximately 50% of the wild-type level. One possible explanation for this may be due to the gene being present on multiple copies of the plasmid.

*C. elegans* has been used as a simple surrogate host for modeling bacterial diseases [21]. It has been shown that on a low nutrient nematode growth medium (NGM)* B. pseudomallei* killed* C. elegans* strain N2 within 3 days and this type of killing is referred to as “slow killing” [22–24]. In our study, wild-type strain Bp-CMS killed 74% of the nematode population at 72 h time point in NGM. Our results are in agreement with published reports that differences in killing efficiency of* C. elegans* occur among wild-type strains of* B. pseudomallei* [23, 25, 26]. For instance, the percentage of killing of worms by various* B. pseudomallei* strains, that is, ATCC23343, EY4, number #40, and KHW, was approximately 50, 60, 75, and 90%, respectively, at 72 h time points [23]. Virulence of* B. pseudomallei* for the nematode is likely to be variable due to the different genetic determinants in the strains.

In the present study, all the constructed mutants were less effective in killing* C. elegans* under slow-killing conditions; that is, twice as many worms survived when fed on mutant strains compared to worms fed on wild-type Bp-CMS after 72 h of coculture. However, there was no significant difference of* C. elegans* killing between the mutants and wild type. Two complemented strains of* BPSL2033::Km* and* BURPS1710A_1419::Km* achieved at least partial restoration of virulence at 72 h coculture, thus suggesting that both genes are most probably involved in bacterial virulence.

The low level attenuation of virulence in the mutants, compared to the wild type, may possibly be due to the following.

- A single gene was insufficient to mediate full killing in the animal model. At least 2 genes are probably required to act together for achieving virulence in* B. pseudomallei.* A double mutant Δ*relA*Δ*spoT* was reported to exhibit significant and severe attenuation in larva of the wax moth* Galleria mellonella* and C57BL/6 in mice models, which was not seen with the single mutant [20].
- *C. elegans* possesses mechanisms to avoid or move away from pathogenic bacteria like* B. pseudomallei*. It has a simple nervous system that consists of 302 neurons that facilitate the identification of molecules, neurons, and circuits involved in their behavior [27]. It uses chemotaxis to find food on the plate and is able to discriminate food both physically based on size and chemically based on taste and olfaction [28]. Worms can modify their olfactory preference via the neurotransmitter serotonin to avoid odours from pathogens and this learning occurs with exposure as short as 4 h [29]. In our study,* C. elegans* was cultivated on* E. coli* OP50-1 that best supports growth, so the worms had already experienced good food, which might have increased their exploratory bahaviour when switching to very bad food such as* B. pseudomallei,* especially in leaving bahaviours. Nonetheless, it is impossible to raise worms on* B. pseudomallei* alone because of the virulence of the organism.

*BPSL2033* is a 428-amino-acid protein with a molecular mass of 46 kDa as a transport-related membrane protein;* BURPS1710A_1419* is a 74-amino-acid protein with a calculated molecular mass of 8 kDa which is a putative lipoprotein. Further bioinformatics analysis suggests that amino acids 23-324 of* BPSL2033* encode a domain belonging to major facilitator superfamily (MFS). MFS transporters are ubiquitous and found in all classes of organisms and in several pathogens such as* Francisella tularensis* [30] and* Legionella pneumophila* [31]. Chatfiled and colleagues [32] have shown that MFS protein plays a role in virulence by promoting bacterial iron-siderophore import.

The Phyre 2 model [33] predicts that* BPSL2033* forms a major facilitator superfamily fold and shares very low sequence identity (16%) to glycerol-3-phosphate transporter protein from* E. coli* with known three-dimensional structure (PDB template: 1pw4A). The results imply that* BPSL2033* might exhibit new structural and/or functional characteristics and further X-ray crystallography analysis may provide valuable information. At present, we postulate that* BPSL2033* transports nutrients (probably glycerol-3-phosphate) that are essential for the replication of* B. pseudomallei*. Database searches with the NCBI blastp tool identified* BURPS1710A_1419*, as a putative lipoprotein within genus level, is diverged from 37 to 82% with no conserved domain identified, as well as a lack of three-dimensional structural information, possibly suggesting that* BURPS1710A_1419* gene product has new or different functional characteristics.

In conclusion, our results suggest that* BPSL2033* and* BURPS1710A_1419* individually are likely to contribute to a minor role in virulence and provide a basis for further characterization of their role in pathogenesis. We hypothesize that the combination effect of both genes can provide a clear virulence role in* B. pseudomallei*.

## Figures and Tables

**Table 1:** Bacterial strains and plasmids used.

Strains or plasmids	Relevant characteristic(s)	Reference
Strains		
*B. pseudomallei *		
Bp-CMS	Clinical isolate from blood; wild type parental strain for generation of insertion mutants	Current study
*BPSL2033::Km*	*BPSL2033::Km* derivative of CMS; Kan^R^	
*BPSL2033::Km* (pC-*BPSL2033*)	*BPSL2033::Km* complemented with pC-*BPSL2033 *plasmid; Kan^R^ Cm^R^	
*BP1026B_I2784::Km*	*BP1026B_I2784::Km* derivative of CMS; Kan^R^	
*BP1026B_I2784::Km* (pC*-BP1026B_I2784*)	*BP1026B_I2784::Km* complemented with pC*-BP1026B_I2784* plasmid; Kan^R^ Cm^R^	
*BP1026B_I2780::Km*	*BP1026B_I2780::Km* derivative of CMS; Kan^R^	
*BP1026B_I2780::Km* (pC-*BP1026B_I2780*)	*BP1026B_I2780::Km* complemented with pC*-BP1026B_I2780* plasmid; Kan^R^ Cm^R^	
*BURPS1106A_A0094::Km*	*BURPS1106A_A0094::Km* derivative of CMS; Kan^R^	
*BURPS1106A_A0094::Km *(pC*-BURPS1106A_A0094*)	*BURPS1106A_A0094 ::Km* complemented with pC*-BURPS1106A_A0094 *plasmid; Kan^R^ Cm^R^	
*BURPS1106A_1131::Km*	*BURPS 1106A_1131::Km* derivative of CMS; Kan^R^	
*BURPS1106A_1131::Km* (pC-*BURPS1106A_1131*)	*BURPS 1106A_1131::Km* complemented with pC*-BURPS1106A_1131* plasmid; Kan^R^ Cm^R^	
*BURPS1710A_1419::Km*	*BURPS1710A_1419::Km* derivative of CMS; Kan^R^	
*BURPS1710A_1419::Km* (pC-BURPS1710A_1419)	*BURPS1710A_1419::Km* complemented in *trans* with pC-*BURPS1710A_1419 *plasmid; Kan^R^ Cm^R^	
*BPSL3147::Km*	*BPSL3147::Km* derivative of CMS; Kan^R^	
*BPSL3147::Km* (pC-*BPSL3147*)	*BPSL3147::Km* complemented with pC-*BPSL314*7 plasmid; Kan^R^ Cm^R^	
*E. coli *		
DH10B	F_ *mcrA *Δ(*mrr-hsdRMS-mcrBC*)*ϕ80lacZ *Δ*M15 *Δ*lacX74 recA1 endA1 araD 139 *Δ(*ara, leu*)*7697 galU galK λ* ^−^ *rpsL nupG *	Prof. Dr. Jin-Town Wang
CC118*λ*pir	Δ(*ara-leu*)* araD *Δ* lacX74 galE galK phoA20 thi-1 rpsE rpoB argE*(*Am*)* recA1 λpir *phage lysogen
S17-1*λ*pir	hsdR recA pro RP4-2 (Tc::Mu; Km::Tn7) (*λ*pir)
OP50-1	A streptomycin-resistant derivative of *E.coli *OP50 used as food source in *C. elegans* cultivation and uninfected control	Kind gifts from Prof. Dr. Sheila Nathan
Plasmid		
pUT-Km	pUT-Km1 derived plasmid, with miniTn5 excised by *Eco*RI, *tnp* excised by *Sal*I, and bla removed by *Apa*LI and then with an insertion of Km resistance cassette from pUC4K into *Pst*I site; *ori*R6K *mob*RP4 Kan^R^ Amp^R^	Chuang et al., 2006 [15]
pGEM-T easy	Cloning vector for PCR cloning; Amp^R^	Promega, USA

**Table 2:** Primers used for PCR and construction of mutants and complemented plasmids.

Gene	Primer name	Nucleotide sequence (5′ to 3′)	Purpose
*BPSL2033 *	2033IF	AGAACTTCGAGCAATTGCTG	Internal PCR
	2033IR	GAGAGATGACGTTCGGTCTT	Internal PCR
	M2033F	GTGAACTGGTACAAAGAAATATCG	Mutant confirmation
	M2033R	CACGTTTCTCGGATAGAGC	Mutant confirmation
	C2033F	CTAGAGCGCGGCCTCGCG	Complementation of *BPSL2033::Km*
	C2033R	CATCACTCGGCGCAATGAGACTG	Complementation of *BPSL2033::Km*

*BP1026B_I2784 *	2784IF	GTCGAGAGTACGGTGTGTTC	Internal PCR
	2784IR	CCTGCGAAATCCTTATCAC	Internal PCR
	M2784F	CTGTTTTCTAAGCGTCAGAAG	Mutant confirmation
	M2784R	AAATATGCAGGAAATAGCCCG	Mutant confirmation
	C2784F	CCTTCGCGCTGATTTGGT	Complementation of *BP1026B_I2784::Km*
	C2784R	CTACTTCGTAGCTTGATGCGCC	Complementation of *BP1026B_I2784::Km*

*BP1026B_I2780 *	2780IF	AAACCAGAAGGGCGATTTC	Internal PCR
	2780IR	GCGTTCTTTTAAGAATTGGGTAG	Internal PCR
	M2780F	CAGGTACGATTCATGGAACG	Mutant confirmation
	M2780R	GGTATTCCGTGACCTGAATGT	Mutant confirmation
	C2780F	AGGCTCGGAGTAGTAACACTT	Complementation of *BP1026B_I2780::Km*
	C2780R	CTACTGCCTATGCTGGGGTAT	Complementation of *BP1026B_I2780::Km*

*BURPS1106A_A0094 *	94IF	AGTCGGGGGGTACACCTAC	Internal PCR
	94IR	CGACACCGAGGAAAATTTC	Internal PCR
	M94F	GTGAATGTCGATCTTGCG	Mutant confirmation
	M94R	TCAATCTCCAGCGAGCTT	Mutant confirmation
	C94F	TTCTACCAGCGACTTGGC	Complementation of *BURPS1106A_A0094::Km*
	C94R	TCAATCTCCAGCGAGCTT	Complementation of *BURPS1106A_A0094::Km*

*BURPS1106A_1131 *	1131IF	AAGGAGTTGGGTACGTCG	Internal PCR
	1131R	CCCTTGTGCCATTGATAG	Internal PCR
	pUT-R	TTTGAGTGACACAGGAACAC	Mutant confirmation
	C1131F	GTGGTTCAGCCAGGCACG	Complementation of *BURPS1106A_1131::Km*
	C1131R	GTATGTGTCGTCCGCATTTG	Complementation of *BURPS1106A_1131::Km*

*BURPS1710A_1419 *	1419IF	GCGATAGCGATTGGAAAACT	Internal PCR
	1419IR	GAATCCAGACCCATTCCGT	Internal PCR
	KmF	ATGAGCCATATTCAACGGGA	Mutant confirmation
	C1419F	TGCACAAGCTGTTCAAATG	Complementation of *BURPS1710A_1419::Km*
	C1419R	TTAAGGCTTGGGTGCAAG	Complementation of *BURPS1710A_1419::Km*

*BPSL3147 *	3147IF	GACCAGTACGCGCTCAAG	Internal PCR
	3147IR	GAACGAATACTTGTCGATCG	Internal PCR
	M3147F	GATGTACACGTTCAACGACAAG	Mutant confirmation
	M3147R	CTCTTCCGGCATCTCGTA	Mutant confirmation
	C3147F	TTCAGGGTTACGAAGCGAAG	Complementation of *BPSL3147::Km*
	C3147R	TCAGTGCAGCCGGATGCTCG	Complementation of *BPSL3147::Km*
